# Genetic and Environmental Factors Affecting Seed Germination

**DOI:** 10.3390/plants12244106

**Published:** 2023-12-08

**Authors:** Božena Šerá, František Hnilička

**Affiliations:** 1 Department of Environmental Ecology and Landscape Management, Faculty of Natural Sciences, Comenius University in Bratislava, 84215 Bratislava, Slovakia; 2 Faculty of Agrobiology, Food and Natural Resources, Czech University of Life Sciences Prague, Kamycka 121, 16500 Prague, Czech Republic; hnilicka@af.czu.cz

Plants have evolved various strategies allowing them to be successful in heterogeneous habitats, including the number and size of the seeds they produce, mechanisms for their dispersal, seed dormancy, seed vigor, seed germination, etc. Seed germination is important for generative reproduction, the plant life cycle and population growth. Studying the factors influencing seed germination is one of the most useful applications in terms of the morphology and physiology plants, molecular biology, genetics and ecology.

This Special Issue provides an overview of the latest advances in the field of seed germination stimulating/influencing. It brings to the forefront research contributions from various scientific fields, such as basic research, plant protection, agriculture, forestry and ecology. The presented articles can be divided into the following six topics, which are directly related to influencing seed germination (Figure 1).

**Seed Quality**

Arceo-Gómez et al. [1] studied the seeds of *Enterolobium cyclocarpum* and *Piscidia piscipula*, Fabaceae tree species from the Yucatan Peninsula (Mexico). They used fresh seeds to determine seed permeability and imbibition rate, they further analyzed seed viability using the tetrazolium test, and applied mechanical scarification and boiler shocks for 5 s, 10 s and 15 s treatments. The presence physical dormancy in the seeds was confirmed in both species; the seeds became permeable and displayed high germination following the application of pre-germinative treatments. Seed permeability and the imbibition rate in *E. cyclocarpum* were low. The highest germination in *E. cyclocarpum* was seen during the mechanical scarification, at 92%, while in *P. piscipula*, this parameter was seen in the 10 s boiling water treatment, at 76.0%. *E. cyclocarpum* had the highest germination percentage during the mechanical scarification, while *P. piscipula* showed the highest germination percentage following thermal shocks with boiling water for 10 s. The present research seeks to promote the sustainable use of *E. cyclocarpum* and *P. piscipula,* contribute to the creation of effective conservation strategies and enhance their biotechnological applications.

Hamdiah et al. [2] were interested in the biological characteristics of the seeds of nine endemic taxa *Boswellia* (Burseraceae) tree species from Socotra Island (Yemen). They tested seed germination rates in controlled experiments for two subsequent years. They noted a large variation in seed germination (relatively high in *Boswellia socotrana*), but half of the species showed a relatively low mean daily germination. Regarding the protection of these species, they summarized that all of the species they tested harbor the potential for in situ conservation (in combination with the seed germination in local nurseries) and suggested more detailed studies of their seeds in the future.

**Physical, Chemical and Biological Factors**

Article [3] summarizes the results of a 12-year-long research project on the viability and radioresistance of greater plantain (*Plantago major* L.) seed progenies growing in the East Ural Radioactive Trace zone and in control (nonradioactive) areas, with a consideration of the variability of the weather conditions in this area. Absorbed dose rates of *P. major* parental plants in the pollution gradient were 14.5–165.9 µGy h^−1^; the quality of the seed progenies was evaluated using seed weight, survival rate and the length of seedling’s roots. Interannual variability in the parameters they studied was high, and their ranges overlapped between contaminated groups and control groups in most cases. In the control groups of seeds, 88.9% of correlations were negative, whereas in the contaminated groups, 78.5% were positive. Shimalina et al. [3] indicated that, during research on radiobiological effects in natural populations of living organisms, it is necessary to take into account that the observed effects vary among different years.

In their study, Faundez et al. [4] analyzed the effect of soil matric potential on seed germination and initial recruitment in the sclerophyllous species *Prosopis chilensis*, *Quillaja saponaria* and *Cryptocarya alba,* from contrasting geographic origins, under different matric potentials (i.e., 0, −6, −33, −750 and −1250 kPa). *P. chilensis* seeds stopped germinating under a soil matric potential close to −1200 kPa, whereas in *Q. saponaria* and *C. alba,* the complete inhibition of germination occurred under −1000 kPa. Their results showed the clear effect of seed source on germination capacity regardless of the soil matric potential, thus confirming the relevance of the seed source in the regeneration of the species under study.

**Seed Pre-treatment**

Bednařík et al. [5] found that the fermentation of immature seed material from *Tilia coradata*, consisting of moistening, a 7-day anaerobic phase and a 10–30-day aerobic phase, appeared to be an effective pre-sowing treatment. It was more advantageous than the classic warm–cold stratification of fully matured seed, or the sowing of immature seed material without any pre-sowing treatments. The seeds were non-dormant after fermentation and were thus ready for sowing in the spring following collection, which may shorten the time needed to prepare seeds for sowing by one year.

Non-thermal plasma (NTP) technologies are being developed for agricultural uses and do not cause damage to heat-sensitive biological systems. This seed technology has shown the potential to improve agronomic seed quality by enhancing germination and promoting plant growth. However, there is almost no information about the effect of NTP pretreatment on the emergence of wild plant species’ seedlings. Meadow restoration and creation projects have faced a lack of local seed diversity due to the limited availability of seed sources. Therefore, the study by Gudyniene et al. [6] aimed to evaluate the effect of NTP on the emergence of 17 perennial plant seeds (*Anthyllis vulneraria*, *Campanula bononiensis*, *C. glomerata*, *Dianthus borbasii*, *Filipendula vulgaris*, *Galium boreale*, *Helianthemum nummularium*, *Polemonium caeruleum*, *Prunella grandiflora*, *P. vulgaris*, *Ranunculus acris*, *Rhinanthus serotinus*, *Salvia pratensis*, *Scabiosa columbaria*, *S. ochroleuca*, *Veronica teucrium* and *Vicia pisiformis*) originating from local meadows in Lithuania and compare it to the cold stratification pretreatment. Their observations showed that NTP treatment led to increases in 11.8% of the examined plant seeds, whereas stratification demonstrated a beneficial effect on 41.7% of these species. This study highlights the need to customize NTP pre-treatment methods for specific plant species and to conduct further research to understand the underlying mechanisms.

In the study by Florescu et al. [7], the effect of NTP treatment on sunflower seeds (*Helianthus annuus* L.) has been demonstrated in laboratory conditions and after harvest in the field. The seeds were treated with NTP in a dielectric barrier discharge reactor, operated in air, for 10 min (sinusoidal voltage of 16 kV and amplitude at 50 Hz frequency). After a slow start, plasma-treated seeds developed faster and produced taller seedlings with greater total weight compared to controls. Results obtained from mature plants grown in the field showed a positive effect with respect to capitula size, number of seeds per capitulum and weight per thousand seeds, resulting in a significant increase in crop yield.

Recently, much attention has been paid to the use of NTP and plasma-activated water (PAW) in various areas of biological research. While direct NTP action involves the effects of many highly reactive species with short lifetimes, the use of PAW involves the action of only long-lived particles. In their article, authors Jirešová et al. [8] review the literature describing the composition and properties of PAW prepared via various methods. The effects of PAW on the properties (seed germination, early growth, decontamination) of wheat grains (*Triticum aestivum* L.) were determined in laboratory experiments. The authors also draw attention to an otherwise rather neglected fact: that there were no significant differences between the action of PAW and artificially prepared PAW.

**Management**

The ability of *Oryza sativa* L. to elongate its coleoptiles under oxygen deprivation is a determinant of anaerobic germination tolerance, which is critical for successful direct seeding. Aerial seeding of *O. sativa*, in which seeds are exposed to light on flooded soils during the daytime, is gaining popularity. Therefore, the present study [9] investigated physiological mechanisms underlying anaerobic coleoptile growth under light and dark cycles. Hirano et al. [9] found novel *O. sativa* lines (LG and L202) that have the ability to develop long coleoptiles under oxygen deprivation, equivalent to well-characterized anaerobic germination tolerant lines. Thus, this contribution can have a direct impact on the management of rice fields.

Bottle gourd (*Lagenaria siceraria* (Molina) Standl) is a well-known cucurbit with an active functional ingredient. A two-year field experiment was carried out in study by Malik et al. [10]. The fruits harvested from different crossing periods under different environmental conditions influenced the bottle gourd’s qualitative and biochemical traits, and significant variations were shown among the five crossing period environments. The fourth week of the crossing period during the Kharif season (in India), with a temperature of 31.7 °C, appears to be favorable for high-quality *L. siceraria* seed production. Global temperatures are on the rise due to climate change, and there may be significant implications for both crop yields and the nutritional properties of seeds. Research in this field is important for agriculture in areas which are sensitive to climate change.

**Salt or Drought Stress**

Song et al. [11] studied differences in the morphological and physiological characteristics, seed germination and seedling establishment in response to salt stress between dimorphic seeds in *Suaeda liaotungensis*. Brown seeds had higher ROS levels, POD and CAT activities and betaine content, as well as lower SOD activity and MDA and proline contents, than black seeds. Light and temperature promoted the germination of brown seeds, whereas they had no effect on the germination of black seeds. These findings lay the foundation for understanding the ecological adaptation mechanisms in *S. liaotungensis* seeds.

In their study, Borawska-Jarmułowicz and Mastalerczuk [12] evaluated the effects of drought and root extracts (from *Medicago sativa*) at different pH levels on germination and seedling properties in *Lolium perenne*, *Medicago* × *varia* and *M. sativa* in separate growing trials or in mixtures. A lower concentration of root extract had a beneficial effect on the morphological characteristics (root and leaf length, fresh and dry weight) of seedlings when germination was performed separately for both species. The used root extracts, in combination with acidic pH conditions, limited the germination and growth of *L. perenne* seedlings the most during independent germination. This study concluded that, the application of silicon did not improve seed germination under drought conditions, while the germination of *L. perenne* seeds mixed with *M. sativa* mitigated the negative allelopathic effects of root extracts on the seed germination and morphological characteristics of *L. perenne* seedlings. Based on these results, they have come to the conclusion that it is necessary to pay attention to the selection of species and their proportion in mixtures sown on grasslands where the soils are characterized by a low pH.

**Seed Technology**

Aghbolaghi et al. [13] carried out the encapsulation of the embryogenic callus of *Stipagrostis pennata* (from desert regions of Iran) with the subsequent formation of artificial seeds. The *S. pennata* is a drought-resistant grass, but it has a low capacity for seed production, germination and growth, so it is vulnerable to extinction. The most suitable conditions for the production of artificial seeds were 2.5% sodium alginate with an ion exchange time of 30 min after the germination of the artificial seeds on a Murashige and Skoog culture medium, with zeatin riboside and L-proline in vitro cultures. An analysis of the experimental results showed that the total protein content in zygotic seedlings and seedlings derived from synthetic seeds showed no statistically significant differences between these samples. The results suggest using a temporary immersion bioreactor for shoot micropropagation *S. pennata*.

**Conclusions**

This Special Issue highlights recent breakthroughs and findings in the research of seed germination in agricultural crops, as well as in trees that are important in forestry and in wild species. The articles herein are divided into logical groups so that the readers can orient themselves better.

## Figures and Tables

**Figure 1:**
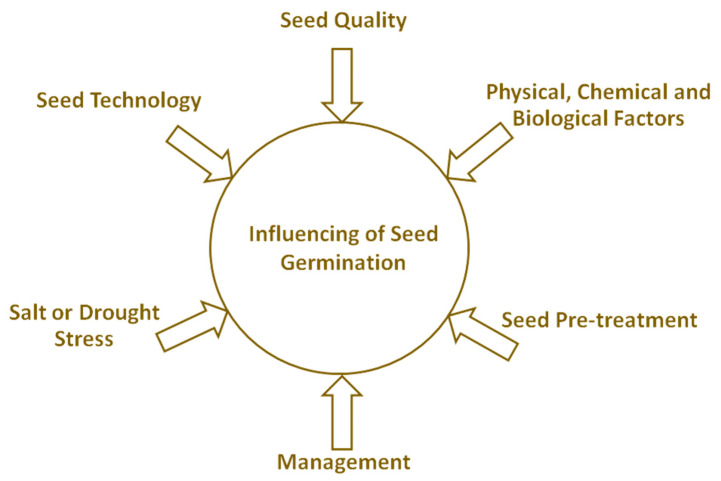
Topics in this Special Issue. Overview of topics and articles related to them: seed quality [1,2], physical and biological factors [3,4], seed pre-treatment [5,6,7,8], management [9,10], salt and drought stress [11,12], and seed technology [13].
